# Toxicity and sublethal effects of two plant allelochemicals on the demographical traits of cotton aphid, *Aphis gossypii* Glover (Hemiptera: Aphididae)

**DOI:** 10.1371/journal.pone.0221646

**Published:** 2019-11-19

**Authors:** Kangsheng Ma, Qiuling Tang, Pingzhuo Liang, Jin Xia, Baizhong Zhang, Xiwu Gao

**Affiliations:** 1 Department of Entomology, China Agricultural University, Beijing, China; 2 College of Resources and Environment, Henan Institute of Science and Technology, Xinxiang, China; Institut Sophia Agrobiotech, FRANCE

## Abstract

Plant allelochemicals are a group of secondary metabolites produced by plants to defend against herbivore. The mortality of two plant allelochemicals (tannic acid and gossypol) on the cotton aphid, *Aphis gossypii* Glover (Hemiptera: Aphididae), were investigated using feeding assays and the sublethal effects were evaluated using the age-stage, two-sex life table approach. Tannic acid and gossypol have deleterious effects on *A*. *gossypii*, and as the concentrations increased, the mortality of cotton aphid increased. The life history traits of *A*. *gossypii* including the developmental duration of each nymph stage, the longevity, oviposition days, total preadult survival rate and adult pre-oviposition period were not significantly affected by sublethal concentration of tannic acid (20 mg/L) and gossypol (50 mg/L), while the population parameters (*r*, *λ* and *R*_0_) were significantly affected by these two plant allelochemicals. Furthermore, tannic acid can increase the pre-adult duration time and TPOP but reduce the fecundity of *A*. *gossypii* significantly compared to the control and gossypol treatment groups. These results are helpful for comprehensively understanding the effects of plant allelochemicals on *A*. *gossypii*.

## Introduction

Plant allelochemicals, sometimes called secondary plant compounds because they are produced as by-products of intermediary metabolism by plant, which may play important roles in defense against insect herbivore [1–3]. Many kinds of plant allelochemicals were deleterious to herbivorous insects [4–7]. For example, isoflavonoids isolated from *Cicer arietinum* can affect the development of *Helicoverpa armigera* larvae [8]. Golawska et al. found that naringenin and quercetin have detrimental effects on the pea aphid, *Acyrthosiphon pisum* Harris [4]. Similarly, Zhang et al. found that the development of *H*. *armigera* was retarded significantly when the 6^th^ instar larvae fed on a sublethal dosage of 2-tridecanone [9].

Tannic acid and gossypol from cotton plants participated in the cotton’s defense system against pathogens and herbivorous insects [10, 11]. Some studies showed that high-gossypol cultivars of cotton plant can negatively affect the development and reproduction of insects, such as *Aphis gossypii* and *Bemisia tabaci* [12, 13]. Moreover, feeding assays with gossypol mixed with artificial diet showed that gossypol has hormetic and detrimental effects on the growth of insects in different concentrations [14, 15]. For example, gossypol was found to have a hormetic effect on the larval growth of *H*. *armigera* at low concentrations, which has otherwise deleterious effect at higher concentrations [14]. Similarly, tannic acid was highly toxic to *Malacosoma disstria* larvae [16] and caused significantly inhibition in the development of herbivorous insects [10, 16, 17]. Although, the effects of tannic acid and gossypol on the growth of several insects have been reported, knowledge about the toxicity and impact of these two plant allelochemicals on *A*. *gossypii*, a destructive insect pest in the cotton fields [18], is still limited.

It is well known that exposure to sublethal concentrations of insecticides could affect insect population dynamics through changed biological and behavioral traits on individuals [19–21]. For plant allelochemicals, although most of these metabolites are high toxic to phytophagous insect [3], the insect pests contact these chemicals with sublethal concentrations in natural environment. Therefore, in this study, we aimed at assessing the potential effects of tannic acid and gossypol on the main life history traits of *A*. *gossypii*.

Life tables is a reliable tool for the prediction of the stage structure and growth of pest populations [22], which can offer a comprehensive description of population dynamics and help illuminate multiple effects of insecticides on insects [23–26]. In our study, the age-stage, two-sex life table was employed for investigating the effects of tannic acid and gossypol on both the life history traits of *A*. *gossypii* individuals and their demographic parameters. The results should be meaningful for understanding the effects of plant allelochemicals on *A*. *gossypii*, and helpful for developing integrated pest management programs for cotton aphid control.

## Materials and methods

### Insects

The strain of *A*. *gossypii* used in this study was collected from cotton fields in the Xinjiang Uygur Autonomous Region of China, was maintained without any insecticide exposure. The aphids were reared on the cotton seedlings, *Gossypium hirsutum* (L.), in controlled conditions of 22 ± 1°C, 65 ± 5% relative humidity, and a photoperiod of 16:8 h (L: D) as described previously [27].

**Ethics approval** No ethics approval was required for this research.

### Chemicals

Tannic acid and gossypol were purchased from Sigma-Aldrich (Sigma-Aldrich, St. Louis, USA). Triton X-100 was obtained from Amresco Inc. (Solon, OH, USA). All other chemicals and solvents used were analytical reagent.

### Feeding assays

The tests were conducted in controlled conditions of 22 ± 1°C, 65 ± 5% relative humidity, and a photoperiod of 16:8 h (L: D). The 0.5 mol/L sterile sucrose solution was used as a liquid artificial diet for the oral delivery of studied plant allelochemicals to *A*. *gossypii* [28]. The tannic acid and gossypol were incorporated into the diet at five concentrations, which were 5, 10, 20, 40, 80 mg/L for tannic acid, and 12.5, 25, 50, 100, 200, 400 mg/L for gossypol. Control diets (without plant allelochemicals) were also included. Sterilized glass tubes that open at both ends were used for in vitro feeding assays, and the details were described in the previous publication [28]. One end of each tube was covered with two layers of parafilm, and 100 μL of the artificial diet containing either tannic acid or gossypol was sandwiched between the two parafilm layers. Thirty healthy apterous adults were gently placed into the tube with a brush and the tube was sealed with a piece of Chinese art paper using solid glue. The aphids were allowed feeding on artificial diet for 72 h and the mortality rate of the aphids was recorded. Each treatment had three replicates.

### Sublethal effects of tannic acid and gossypol on the cotton aphids

To assess the effects of tannic acid and gossypol on the development of *A*. *gossypii*, the sublethal effects on various life history traits and demographic parameters of *A*. *gossypii* were evaluated in this study. Based on the results of preliminary bioassays, 20 mg/L tannic acid and 50 mg/L gossypol were used as a sublethal concentration, respectively. For sublethal assays, apterous adult aphids were placed on artificial diet that containing 20 mg/L of tannic acid or 50 mg/L of gossypol for 24 h. Adults were then removed, and the newborn nymphs were maintained on the diet for a further 24 h. And then, the survival nymphs were placed onto the 20 mm diameter leaf discs, which were placed upside down on agar beds (1.5 mL of 2% agar) in wells of 12-well cell-culture plates and covered with filter paper to prevent escape. At least 35 nymphs were observed individually in each group. The life history traits of aphids, including development time, fecundity, mortality, and longevity were monitored daily. During the reproductive period, the newborn nymphs were counted and removed daily. New cotton leaf discs were replaced every 4 days until the death of the adult.

### Data analysis

For feeding assays, the LC_25_ and LC_50_ values were calculated using a log-probit model by PoLoPlus 2.0 software (LeOra Software, Petaluma, CA). The concentration-mortality relationship (data corrected for control mortality) was considered valid, when there was absence of significant deviation between the observed and the expected data (*P* > 0.05). Mortalities between the treatment and control were compared by 𝜒^2^ using SPSS 19.0 (IBM, Armonk, NY, USA). The life table data for all *A*. *gossypii* individuals in this study were analyzed according to the age-stage, two-sex life table theory [29, 30]. The population parameters, including the intrinsic rate of increase (*r*), finite rate of increase (*λ*), net reproductive rate (*R*_0_), the mean generation time (*T*), age-stage specific survival rates (*s*_*xj*_, where *x* is age and *j* is stage), age-specific survival rate (*l*_*x*_), age-specific fecundity (*m*_*x*_), adult pre-oviposition period (APOP), total preoviposition period (TPOP), reproductive days (Rd) (i.e., the number of days that adult produced offspring), age-specific maternity (*l*_*x*_*m*_*x*_), age-stage specific life expectancy (*e*_*xj*_), reproductive value (*v*_*xj*_), were calculated according to Chi and Liu [30] and Chi [29] by using the computer program TWOSEX-MSChart [31]. The variances and standard errors of the population parameters were estimated using the bootstrap procedure [32] with 100,000 random resampling and the difference of population parameters between control and plant allelochemical treatment groups were compared by using the paired bootstrap test based on the confidence intervals of differences implemented in TWOSEX-MSChart [31, 33, 34]. All graphics were constructed using SigmaPlot 12.0 (Systat Software Inc., San Jose, CA, USA).

## Results

### Toxicities of tannic acid and gossypol against *A*. *gossypii* adults

Acute toxicity of tannic acid and gossypol against the adult cotton aphids was determined via the liquid artificial diet incorporation method. Log-probit regression analysis of concentration-mortality data after 72 h treatment revealed that LC_50_ and LC_25_ values for tannic acid were 41.04 mg/L (95% confidence interval: 27.91–50.69 mg/L) and 23.70 mg/L (95% confidence interval: 10.42–32.76 mg/L) with a regression equation of *Y* = 0.436 + 2.829*X* (*χ*^2^ = 7.54, df = 13, *P* = 0.872) and for gossypol were 314.51 mg/L (95% confidence interval: 126.07–1007.96 mg/L) and 51.49 mg/L (95% confidence interval: 4.00–127.98 mg/L) with a regression equation of *Y* = 2.875 + 0.858*X* (*χ*^2^ = 12.38, df = 16, *P* = 0.718), respectively.

To ensure low mortality, bioassays to examine sublethal effects of tannic acid and gossypol exposure were then conducted at a LC_25_ value of 20 mg/L tannic acid and 50 mg/L gossypol in the artificial diet for 24 h. As expected, the mortalities of adults of the cotton aphid were 15.00% and 16.67% for tannic acid and gossypol at 24 h, respectively, and they were not significantly different to 11.67% and 13.33% of the controls (tannic acid: *χ*^2^ = 0.130, df = 1, *P* = 0.718; gossypol: *χ*^2^ = 0.131, df = 1, *P* = 0.718). The 20 mg/L tannic acid and 50 mg/L gossypol were classified as sublethal concentrations and fitted for subsequent sublethal assays.

### Effects of sublethal concentration of tannic acid and gossypol on *A*. *gossypii*

The effects of both tannic acid and gossypol on the development time, longevity, oviposition days, total preadult survival rate and fecundity of cotton aphids were presented in Table 1. No significant differences among tannic acid treatment, gossypol treatment and the control groups were observed in the developmental duration of each nymph stage, the longevity, oviposition days, total preadult survival rate, and adult pre-oviposition period (APOP). Similarly, no significant differences of pre-adult duration time, total pre-oviposition period (TPOP) and fecundity were found between the control and gossypol treatment groups. However, tannic acid treatment significantly increased the pre-adult duration time (*P* = 0.047) and TPOP (*P* = 0.011) of *A*. *gossypii* compared to the control. When compared to the control, the fecundity of *A*. *gossypii* was significantly reduced after tannic acid treatment (*P* = 0.003), but not significantly affected by gossypol treatment (*P* = 0.526). In addition, a significant difference of fecundity was also observed between tannic acid and gossypol treatment groups (*P* = 0.041) (Table 1).

**Table 1:** Mean (± SE) life history parameters for the *A*. *gossypii* exposed to the sublethal concentration of tannic acid and gossypol in liquid artificial diet.

Life history parameters	Control		Tannic acid		Gossypol	
	N	Mean ± SE ^a^^,^ ^b^	N	Mean ± SE ^a^^,^ ^b^	N	Mean ± SE ^a^^,^ ^b^
First instar nymph (d)	43	1.79 ± 0.08a	28	1.93 ± 0.09a	28	1.82 ± 0.09a
Second instar nymph (d)	39	1.41 ± 0.09a	27	1.59 ± 0.13a	23	1.30 ± 0.12a
Third instar nymph (d)	36	1.58 ± 0.08a	25	1.64 ± 0.11a	23	1.57 ± 0.12a
Forth instar nymph (d)	36	1.39 ± 0.08a	25	1.52 ± 0.12a	22	1.64 ± 0.10a
Pre-adult (d)	36	6.17 ± 0.16b	25	6.64 ± 0.18a	22	6.50 ± 0.16ab
Female adult longevity (d)	36	28.75 ± 1.96a	25	23.56 ± 2.67a	22	25.91 ± 2.33a
Female total longevity (d)	36	34.92 ± 1.95a	25	30.20 ± 2.66a	22	32.41 ± 2.41a
APOP (d)	36	0.36 ± 0.08a	23	0.48 ± 0.12a	22	0.27 ± 0.10a
TPOP (d)	36	6.53 ± 0.14b	23	7.13 ± 0.19a	22	6.77 ± 0.21ab
Oviposition days	36	17.31 ± 1.06a	23	15.57 ± 1.43a	22	17.50 ± 1.51a
Mean total longevity (d)	52	25.12 ± 2.46a	35	22.26 ± 2.87a	40	19.12 ± 2.70a
Total preadult survival	52	0.692 ± 0.064a	35	0.7142 ± 0.076a	40	0.550 ± 0.079a
Fecundity (offspring/individual)	36	36.17 ± 2.37a	25	25.16 ± 2.87b	22	33.73 ± 3.10a

^a^ Standard errors (SE) were estimated by using the bootstrap technique with 100,000 re-samplings

^b^ Difference between treatments were compared with paired bootstrap test. The means in same row followed by different lowercase letters indicate significant differences between treatments (*P* < 0.05).

The effects of tannic acid and gossypol on the population growth parameters were estimated with bootstrap methods based on the life table, and the results are presented in Table 2. When compared to the control group, the intrinsic rate of increase (*r*), the finite rate of increase (*λ*) and the net reproductive rate (*R*_0_) were significantly reduced by tannic acid (*P* = 0.041, 0.042 and 0.044 for *r*, *λ* and *R*_0_, respectively) and gossypol (*P* = 0.041, 0.049 and 0.045 for *r*, *λ* and *R*_0_, respectively) treatments, while no significant differences of *r*, *λ* and *R*_0_ were found between tannic acid and gossypol treatments (*P* = 0.955, 0.956 and 0.887 for *r*, *λ* and *R*_0_, respectively). Among tannic acid treatment, gossypol treatment and control groups, no significant differences of mean generation time (*T*) were found (*P* > 0.05). Minimal value for the gross reproduction rate (GRR) of *A*. *gossypii* was observed in tannic acid treatment (33.66 ± 2.01 offspring/individual), which was significantly different from the control (42.25 ± 1.45 offspring/individual; *P* < 0.001) and gossypol (41.38 ± 2.62 offspring/individual; *P* = 0.020), while no significant difference of GRR was found between the control and gossypol treatment (*P* = 0.768).

**Table 2:** Effects of tannic acid and gossypol on population parameters (mean ± SE) of *A*. *gossypii*.

Population parameters ^a^	Mean ± SE ^b^^,^ ^c^		
	Control	Tannic acid	Gossypol
*r* (d^−1^)	0.243 ± 0.011a	0.212 ± 0.013b	0.210 ± 0.015b
*λ* (d^−1)^	1.276 ± 0.014a	1.236 ± 0.016b	1.234 ± 0.019b
*R*_0_ (offspring/individual)	25.050 ± 2.825a	17.967 ± 2.783b	18.546 ± 3.136b
*T* (d)	13.221 ± 0.265a	13.601 ± 0.361a	13.829 ± 0.516a
GRR (offspring/individual)	42.25 ± 1.45a	33.66 ± 2.01b	41.38 ± 2.62a

^a^ *r*, intrinsic rate of increase; *λ*, finite rate of increase; *R*_0_, net reproductive rate; *T*, mean generation time; GRR, gross reproduction rate.

^b^ Standard errors between two treatments were estimated by using the bootstrap technique with 100,000 re-samplings.

^c^ Difference between treatments was evaluated by using a paired bootstrap test. The means in same row followed by different lowercase letters indicate significant differences between treatments (*P* < 0.05).

**Fig 1:**
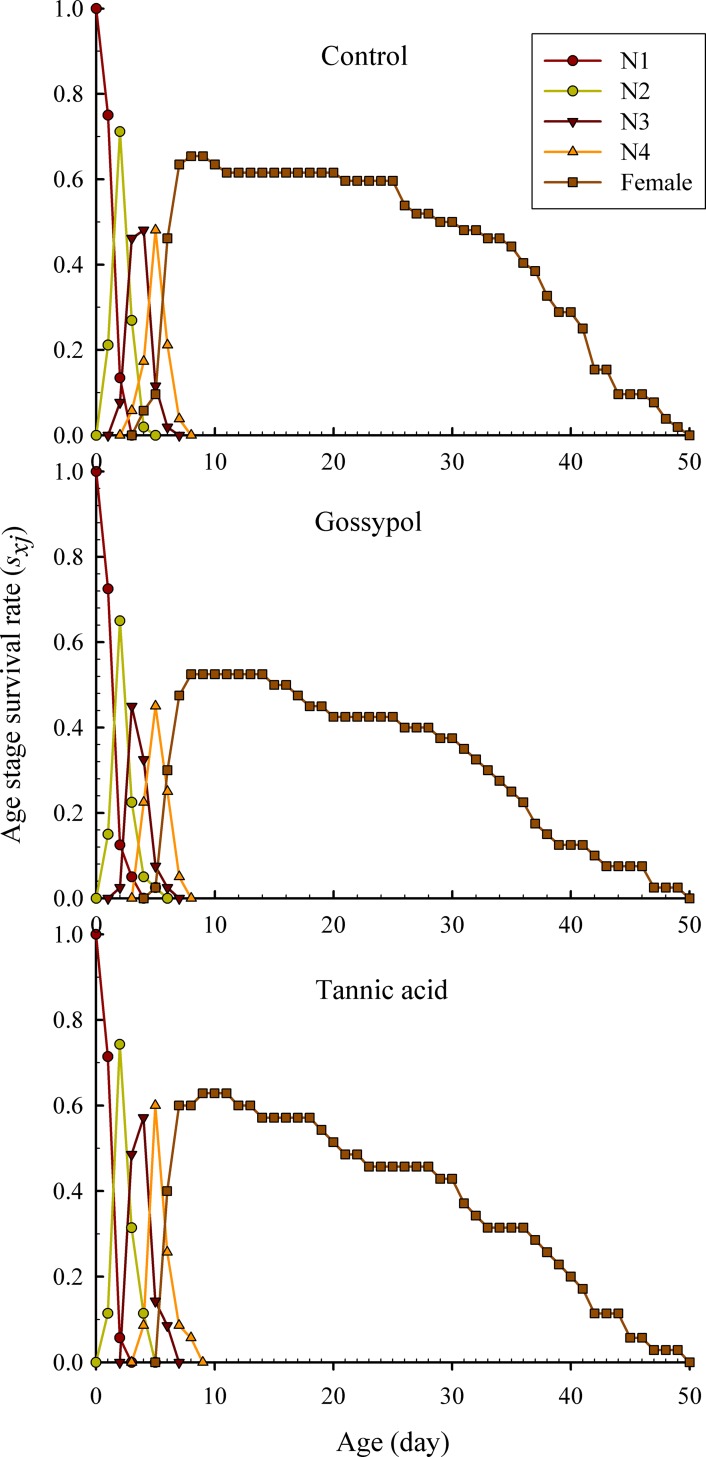
Age-stage survival rate (*s*_*xj*_) of *A*. *gossypii* populations following 24 h treated with 20 mg/L tannic acid and 50 mg/L gossypol.

**Fig 2:**
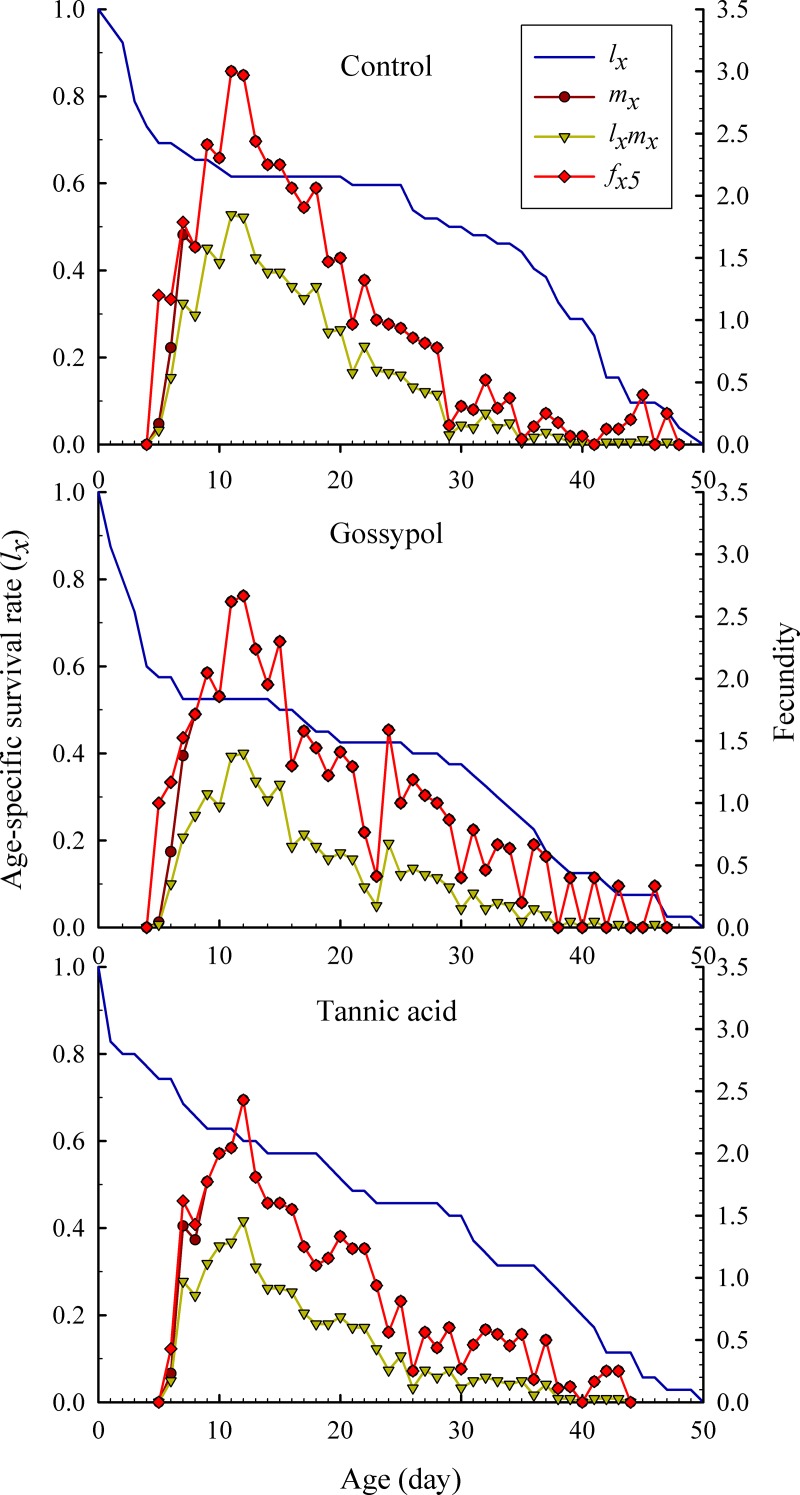
Effects of 20 mg/L tannic acid and 50 mg/L gossypol on the age-specific survival rates (*l*_*x*_), the age-specific fecundity of total population (*m*_*x*_), the age-specific maternity (*l*_*x*_*m*_*x*_), and the age-stage specific fecundity (*f*_*x5*_) of the female adult stage of *A*. *gossypii* populations.

The age-stage specific survival rate (*s*_*xj*_) is the probability a newborn individual will survive to age *x* and stage *j* (Fig 1). Owing to the variable developmental rates among individuals, significantly overlaps between different life stages were observed for both the control group and the plant allelochemicals treatment groups (Fig 1). The age-specific survival rate (*l*_*x*_) is a simplification of *s*_*xj*_ without accounting for the stage differentiation. In this study, the curve of *l*_*x*_ significantly decreased in the plant allelochemicals-treated groups compared to the control group (Fig 2). The highest unique age-specific fecundity peak of the control group (3.007 offspring) occurred at the age of day 11 in the fecundity curve of *m*_*x*_, while the highest fecundity peak of the gossypol (2.688 offspring) and tannic acid (2.431 offspring) treatment groups were observed at the age of day 12 (Fig 2). These results showed that the probability that a newborn nymph would survive to the adult stage decreased in plant allelochemicals treatment groups in comparison with the control group. In addition, we found that gossypol has a lower survival rate of *l*_*x*_ than that of tannic acid (Fig 2), as contrary, tannic acid has a lower fecundity of cotton aphids than that of gossypol (Fig 2).

**Fig 3:**
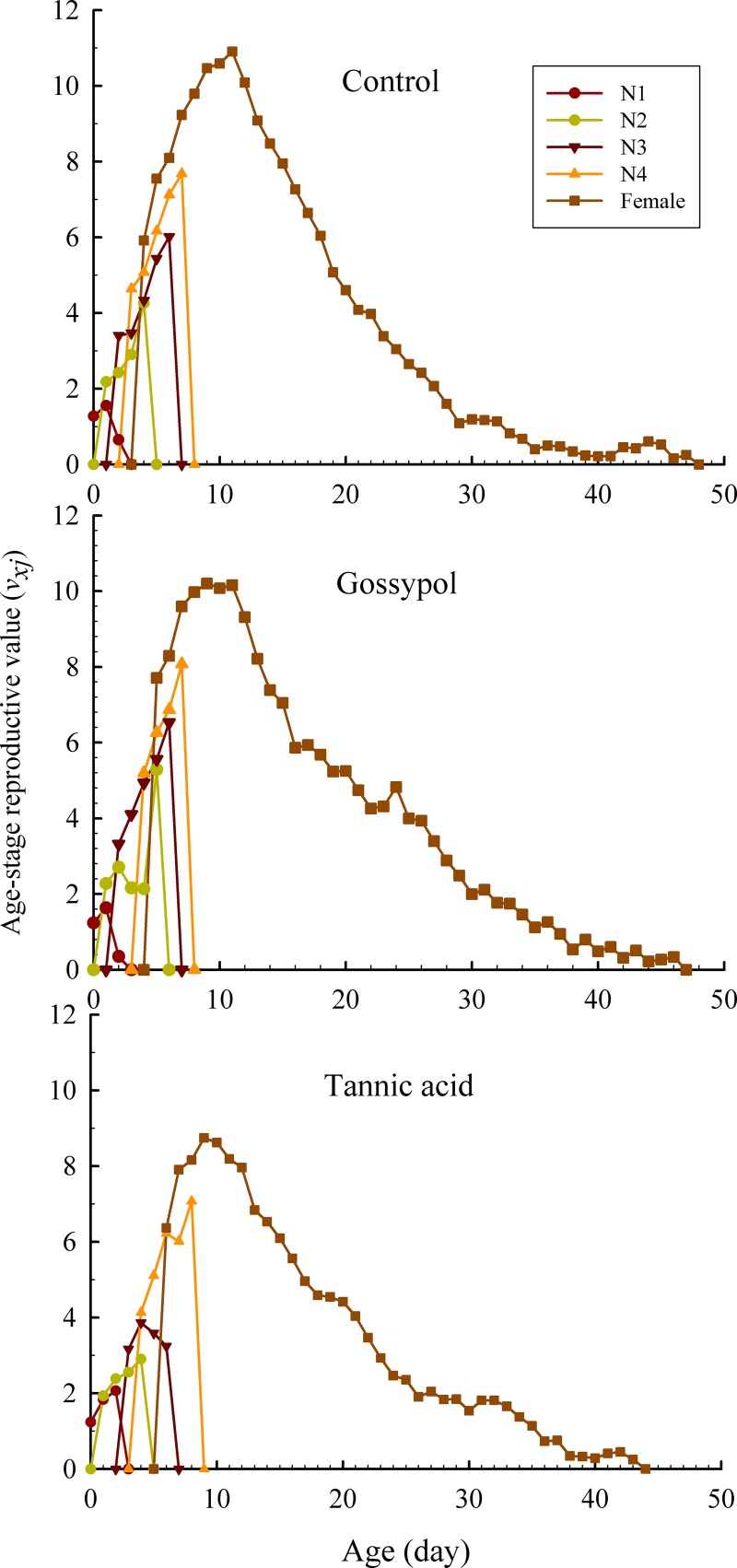
Age-stage reproductive value (*v*_*xj*_) of *A*. *gossypii* populations following 24 h treated with 20 mg/L tannic acid and 50 mg/L gossypol.

The reproductive value (*v*_*xj*_) is the contribution an individual of age *x* and stage *j* will make to the future population of *A*. *gossypii* (Fig 3). The maximum *v*_*xj*_ noted in tannic acid (8.74/day) and gossypol (10.22/day) treatments both occurred at age 9 day, which were lower than that noted in the control occurred later at age 11 day (10.90/day) (Fig 3).

## Discussion

Tannic acid and gossypol are two kinds of secondary metabolites (allelochemical) produced by cotton plants, which involve in the defense system of cotton plants against pathogens and insect herbivores [6, 11, 15, 35]. Both tannic acid and gossypol are toxic to many organisms and feeding of these two chemicals can adversely affect the development of insects [14–16, 36]. For example, when fed on gossypol contained diets the larval pupal weights and survival of *Helicoverpa zea* were significantly reduced compared with the control diets groups [6]. Furthermore, our previous study demonstrated that the developmental periods of *A*. *gossypii* were significantly prolonged and the mean relative growth rates were markedly reduced when cotton aphids fed on spider mites infested cotton plants that contained high levels gossypol and condensed tannins [37]. Therefore, the objective of this study was to evaluate the effects of these two plant allelochemicals on *A*. *gossypii* systemically.

Gossypol is a polyphenolic secondary metabolite and is toxic to many organisms [7, 11]. Stipanovic et al. found that gossypol can adversely affect the survival of *H*. *zea* [6]. In addition, high concentrations of all forms of gossypol reduced the survival and pupal weights for larvae of *Helicoverpa virescens* [35]. In this study, the results of toxicity tests demonstrated that gossypol are toxic to *A*. *gossypii* and fed on high concentration of gossypol led to a higher mortality, similar results were obtained by Peng et al. that the mortality of adults of cotton aphids increased with increasing gossypol concentrations [15]. Du et al found that the aphids fed on the high gossypol cultivar displayed significantly shorter adult longevity and lower fecundity than that of low and medium gossypol cultivars [12]. In the present study, although no significantly difference was observed, we also observed a reduction of longevity and fecundity in gossypol feeding group compared with the control (Table 1).

It is well known that tannins, including tannic acid, can defend plants against insect herbivores by deterrence and/or toxicity [38]. For example, as little as 0.5% tannic acid could cause a significantly reduction in relative growth rate of *Malacosoma disstria* [16]. Our results indicated that tannic acid could cause the death of cotton aphids, and high concentration of tannic acid resulted in significant increase of the mortality, this is in accordance with the fact that negative effects of tannins on both insect and vertebrate herbivores depend on high concentration [39].

The data in life table exhibited that both tannic acid and gossypol had negative effects on cotton aphids, the values of *r*, *λ* and *R*_0_ were significantly reduced and the development time was prolonged at some extent in tannic acid and gossypol treatment groups compared to the control group (Table 1 and Table 2). Similar results were observed in pea aphid that high concentrations of flavonoids increased the developmental time and reduced the intrinsic rate (*r*) [4]. However, this is inconsistent with the results of Yousaf et al. [40], which showed that exposure of F_0_ generation of *A*. *gossypii* to 25 ppm cucurbitacin B only significantly decreased the net reproductive rate (*R*_0_) of F_1_ generation, while other demographic traits of F_1_ (*r*, *T*, and *λ*) were not significantly reduced. Moreover, from the life table we found that tannic acid had a greater effect on cotton aphids than gossypol (Table 1 and Table 2). For instance, the cotton aphid adults that fed on tannic acid exhibited a significantly reduction of fecundity than that of gossypol and control groups (Table 1). Combining the toxicity bioassay results that cotton aphids are more susceptible to tannic acid than gossypol, the cause of this difference may be due to the difference of the toxicity of these two plant allelochemicals to *A*. *gossypii*.

Cotton aphid is one of the most destructive sucking pests on cotton and numerous agriculture crops that causes damage through direct feeding and virus transmission [18, 41]. The control of this pest mainly relies on the application of chemical insecticides in China [42]. However, our previous studies demonstrated that *A*. *gossypii* has evolved very high levels resistance to many types of insecticides [43–48]. Therefore, it is necessary to develop more environmentally friendly control strategies, such as biological control [49–52], biopesticides [53–56], and plant resistance [57, 58]. Our results demonstrate that tannic acid and gossypol have detrimental effect on cotton aphids and these two plant allelochemicals have potential for this pest control. These results are meaningful for understanding the potential plant-aphid interactions, and helpful for developing integrated pest management programs of cotton aphids.
